# Effects of bovine whey protein on exercise-induced gut permeability in healthy adults: a randomised controlled trial

**DOI:** 10.1007/s00421-024-05423-4

**Published:** 2024-02-22

**Authors:** Dulantha Ulluwishewa, Grayson Nicholls, Harold Henderson, Daniel Bernstein, Karl Fraser, Matthew P. G. Barnett, Matthew J. Barnes

**Affiliations:** 1 AgResearch, Te Ohu Rangahau Kai, Palmerston North, New Zealand; 2 School of Sport, Exercise and Nutrition, Massey University, Palmerston North, New Zealand; 3 AgResearch, Ruakura Research Centre, Hamilton, New Zealand; 4 Riddet Institute, Palmerston North, New Zealand

**Keywords:** Intestinal barrier, Exercise, Dairy protein

## Abstract

**Purpose:**

Intestinal permeability is a critical component of gut barrier function. Barrier dysfunction can be triggered by certain stressors such as exercise, and if left unmanaged can lead to local and systemic disorders. The aim of this study was to investigate the effects of a specific whey protein fraction in alleviating exercise-induced gut permeability as assessed by recovery of lactulose/rhamnose (L/R) and lactulose/mannitol (L/M) urinary probes.

**Methods:**

Eight males and eight females (aged 18–50) completed two arms of a double-blind, placebo-controlled, crossover study. For each arm participants performed two baseline intestinal permeability assessments, following which they consumed the treatment (2 g/day of milk powder containing 200 mg of whey protein) or placebo (2 g/day of milk powder) for 14 days, before performing a post-exercise permeability assessment. The exercise protocol involved a 20-min run at 80% of maximal oxygen uptake on a 1% incline.

**Results:**

Mixed model analysis revealed an increase in L/R (23%; *P* < 0.001) and L/M (20%; *P* < 0.01) recovery following exercise. However, there was no treatment or treatment × exercise effect.

**Conclusion:**

The exercise protocol utilised in our study induces gut permeability. However, consuming whey protein, at the dose and timing prescribed, is not able to mitigate this effect.

## Introduction

As one of the largest interfaces separating the internal milieu from the external environment, the intestinal surface is continuously exposed to large amounts of food, chemicals, bacteria, and other potentially harmful antigens. Thus, the human intestine plays a critical role as a barrier, preventing unwanted compounds from entering the body, all the while allowing absorption of nutrients from the diet (Bischoff et al. 2014). Proper regulation of the intestinal barrier is, therefore, vital for optimal health. Usually, only small amounts of antigens enter the intestinal mucosa and interact with local and systemic immune systems, allowing for controlled activation of the immune system. However, if barrier function is dysregulated, increased intestinal permeability leads to abnormally high antigen exposure, resulting in a disruption of the balance between antigen exposure and immune activation. The disruption of the intestinal barrier and increased intestinal permeability is associated with chronic intestinal conditions (such as celiac disease, inflammatory bowel disease, and irritable bowel syndrome), as well as pathological or inflammatory states such as obesity, diabetes and rheumatoid arthritis (Schoultz and Keita 2020). Even in healthy individuals, increased gut permeability can be caused by certain stressors, leading to the passage of unwanted luminal antigens via the epithelium (Camilleri 2019). This can result in an inflammatory cascade that exacerbates the loss of barrier function, and if left unmanaged, can lead to poor digestive or systemic health conditions.

Strenuous physical exercise is an example of a stressor that can lead to barrier dysfunction (Lambert 2008; van Wijck et al. 2012a). During exercise, blood flow is redistributed to the organs with increased activity (i.e. heart, lungs, active muscles), thus reducing blood supply to the splanchnic region. The resulting gastrointestinal ischaemia and the subsequent reperfusion can compromise intestinal barrier function via hypoxia, inflammation, and the formation of reactive oxygen species, leading to increased intestinal permeability. However, this loss of barrier function can be alleviated by the consumption of specific dietary supplements (Shing et al. 2014; March et al. 2017; Pugh et al. 2017). Dairy proteins, for example, are rich in bioactive compounds, and hence are being used in the development of functional foods that support gut health (Korhonen 2009). One commercially available supplement rich in dairy protein is bovine colostrum. Bovine colostrum is the earliest milk produced after giving birth and is compositionally distinct to mature milk (Playford and Weiser 2021). It contains higher levels of protein than mature milk and is rich in bioactive whey proteins such as lactoferrin and lactoperoxidase. Supplementation with colostrum has been shown to mitigate the increase in intestinal permeability caused by exercise (March et al. 2017; Marchbank et al. 2011) or non-steroidal anti-inflammatory drugs (Playford et al. 2001).

Another commercially available bovine-milk-based supplement with potential barrier enhancing properties is a milk protein extract containing all whey proteins with an isoelectric point > 6.8 (Ulluwishewa et al. 2022). The supplement, henceforth referred to as ‘whey protein’, or simply as ‘whey’, is made up of mainly lactoferrin and lactoperoxidase (> 50% w/w), but also contains at least 50 other proteins, including bioactive proteins such as angiogenin and immunoglobulins. While whey protein is currently marketed for its anti-inflammatory and antioxidative properties, its ability to regulate intestinal barrier function is largely unexplored. Recently, we showed that whey protein improves intestinal barrier integrity, and mitigates immune-mediated barrier dysfunction, in an in vitro model of the intestinal epithelium (Ulluwishewa et al. 2022). In this study, we aimed to determine whether consumption of whey protein can mitigate exercise-stress-induced gut permeability. To this end, we carried out a randomised crossover double-blind placebo-controlled human intervention trial in healthy male and female participants.

## Methods

### Participants

Eighteen healthy males and females, aged between 18 and 50 years old, were recruited to participate in the study. Prior to recruitment, a power calculation was carried out in G*Power (v 3.1.9.4) for a repeated measures design with power = 0.80, *α* = 0.05, *n* = 18. Based on this calculation, an effect size (treatment difference divided by standard deviation) of 1.0 was expected. This is higher than the effect size (ES = 0.6) reported by Pugh et al. (2017), but well below the effect size (ES = 4.05) reported by March et al. (2017), who used similar protocols to investigate the effects of dietary interventions on intestinal permeability.

The participants were non-smokers, without a history of gastrointestinal disorder or surgery, with no known or suspected allergy to dairy products, starch, gluten, or artificial sweeteners, and had not consumed performance enhancing supplements in the 6 months prior to recruitment. To ensure participants had the relevant level of cardiovascular fitness, males with a maximal oxygen uptake ($\dot{V}$O_2 max_) below 50 mL/kg/min, or females with a $\dot{V}$O_2 max_ below 35 mL/kg/min, were excluded from the study. Other exclusion criteria included not being vaccinated against COVID-19, participating in another research trial, and having an injury or medical condition that could be made worse by exercising. Females were excluded if they were pregnant, or did not have a regular menstrual cycle. To ensure that gut permeability was not influenced by the menstrual cycle, females tracked their menstrual cycle, and data collection was timed such that female participants undertook their 20-min exercise trial during the follicular phase. For the 14 days prior to the start of the trial, participants were free of illness symptoms, and had not used non-steroidal anti-inflammatory drugs (including oral consumption, or topical use). Participants were instructed to refrain from consuming dairy protein supplements (including whey and/or casein protein powders and bars), non-steroidal anti-inflammatory drugs and taking part in unaccustomed or strenuous exercise from 48 h before baseline intestinal permeability measures, until the last measures were made after the 14-day supplementation period. In addition, participants were prohibited from consuming alcohol and undertaking exercise, other than normal walking, in the 48 h before visits where exercise was completed.

### Ethical approval

Ethical approval was obtained from the Central Health and Disability Ethics Committee, New Zealand (EXP11280). The study was conducted in accordance with the Declaration of Helsinki, and was prospectively registered with the Australian New Zealand Clinical Trials Register (ACTRN12621001560886). Participants provided written informed consent prior to completing the familiarisation session.

### Study design

**Fig. 1:**
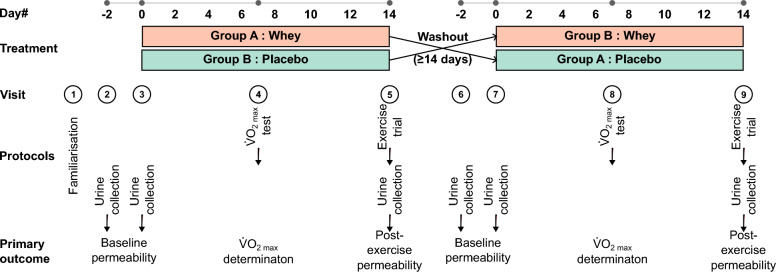
Schematic of clinical study design. Each subject undertook a double-blind crossover trial protocol. Healthy male and female participants supplemented with whey or the placebo for 14 days with a washout period (minimum 14 days) between study arms. The schedule used to determine the $\dot{V}$O_2 max_ that undertake the exercise trials to induce gut permeability and carry out urine collections for gut permeability assessments is shown

To investigate the effects of whey on exercise-induced permeability, participants completed a randomised, double-blind, placebo-controlled, crossover protocol (Fig. 1). Participants were randomly assigned to consume either 200 mg/day whey or placebo for 14 days. Baseline intestinal permeability assessments (pre-supplementation) were carried out on day −2 and day 0 of each arm to confirm a stable baseline. $\dot{V}$O_2 max_ and 80% running speed were determined on day 7 of each arm in preparation for the exercise trial on day 14. Post-supplementation permeability assessments were carried out following the day 14 exercise trial (post-exercise permeability) of both arms. Following a ≥ 14-day washout period, each participant received the alternate treatment, and all procedures were repeated. On the first arm of the study, participants were required to complete a 24-h food diary on the day before the exercise trial and repeat this diet for the second arm of the study. The timing of sampling was based on published studies that used a similar protocol to demonstrate protective effects of dietary components on intestinal permeability (Mahmood et al. 2007; March et al. 2017; Marchbank et al. 2011, 2008; Playford et al. 2001, 2021).

### Preliminary visit

At least 1 week before their first trial, participants attended a familiarisation session during which they provided written, informed consent, completed a health screening questionnaire and resting heart rate, blood pressure, height and weight were recorded. Their $\dot{V}$O_2 max_ was then assessed to ensure they met the required fitness criteria (excellent cardiovascular fitness), to familiarise them with the tests used in the study and to identify appropriate submaximal increments for use in determining running velocity at 80% $\dot{V}$O_2 max_.

### Intervention

At the start of each arm of the study, participants were provided with 14 easy tear stick sachets containing 2 g net weight of dry powder. The sachets were provided by Quantec Ltd., Hamilton, New Zealand. For the whey treatment, the sachets contained (in order of decreasing concentration): skim milk powder, whole milk powder, whey (200 mg), dextrose, and monk fruit extract. The placebo was identical to the treatment with the exception of whey, of which there was none. During the 14-day supplementation period, participants consumed one stick sachet per day on its own or with breakfast. Participants could choose to either pour the contents directly into their mouth and ingest them, or stir the contents of the sachet into a cold or tepid beverage (< 40 °C) and drink. Compliance was verbally confirmed by the participants prior to the day 14 exercise trial.

The whey powder used in this study is commercially available (‘IDP^®^’) and was industrially produced as described previously (Ulluwishewa et al. 2022) by Quantec Ltd (Hamilton, New Zealand). Briefly, water, fat, lactose, and many of the major milk proteins (including casein, α-lactalbumin, and β-lactoglobulin) were removed from milk, resulting in a protein fraction enriched in minor bioactive whey proteins. The final product was a free-flow beige powder (≥ 90% protein) containing all bovine milk whey proteins with an isoelectric point > 6.8, in the same ratios as found natively in bovine milk. While the commercial product is known to contain over 50 proteins, information on only seven has been made publicly available. These include lactoferrin (> 40% w/w), lactoperoxidase (> 18% w/w), angiogenin (~ 10% w/w), immunoglobulin heavy chain (~ 3% w/w), ribonuclease-4 (~ 3% w/w), sulfhydryl oxidase (~ 0.5% w/w), and lysosomal alpha-mannosidase (~ 0.2% w/w) (Data supplied by Quantec Ltd.).

### $\dot{V}$O_2 max_ and running velocity determination

Participants completed four 4-min submaximal stages (e.g. 9, 11, 13, 15 km/h) on a motorised treadmill (True Fitness Technology Inc, Missouri, USA) set at a 1% gradient. After the last submaximal stage, velocity was increased by 1 km/h every minute until volitional exhaustion. Expired gas was measured continuously using an online gas analysis system (Quark CPET, Cosmed, Rome, Italy). The relationship between oxygen consumption and submaximal running velocity was used to formulate a linear regression equation. This equation, in conjunction with the absolute $\dot{V}$O_2 max_ (L/min) value, was used to calculate running velocity at 80% $\dot{V}$O_2 max_ for each participant.

### Exercise trial

On day 14 of each arm of the study, participants returned to the laboratory after an overnight fast of at least 10 h. A heart rate strap (Garmin Ltd, Switzerland) was fitted, and pre-run gut comfort was recorded on a scale of 1 (no discomfort) to 7 (very severe discomfort); this was repeated 10 min into the run and at the end of the run. Participants then ran on a treadmill, with a 1% gradient, for 20 min at the previously determined 80% $\dot{V}$O_2 max_ velocity. Expired respiratory gases and heart rate were recorded continuously throughout the run and 1-min averages were calculated each 5 min and used for analysis. Rating of perceived exertion (Borg 1982) (RPE) was assessed every 5 min during the run. After the run, participants completed a modified version of the Gastrointestinal Symptoms Rating Scale (Svedlund et al. 1988). Instead of rating the intensity, frequency and duration of the symptoms, they were simply asked whether they currently had any of the eleven symptoms by answering “yes” or “no”. Questions related to stool quality were excluded and a question related to the need to urinate was added.

### Urine collection

Lactulose, mannitol, and rhamnose were used as probe molecules to determine the level of intestinal permeability in study participants (Camilleri 2019). These sugars are used because they are non-metabolisable. Lactulose, mannitol and rhamnose for human consumption were obtained from Glentham Life Sciences (United Kingdom). On all occasions where urine was collected, participants fasted overnight before attending the laboratory. To ensure participants were hydrated, they were encouraged to consume water regularly during the overnight fast, and were provided with a 700 mL bottle of water to consume prior to attending the laboratory. Following the overnight fast, either immediately on presentation to the laboratory (baseline permeability) or after the 20-min run (post-exercise permeability), participants emptied their bladders and drank a sugar-probe solution containing 5 g lactulose (disaccharide probe), 2 g mannitol, and 1 g rhamnose (alternative monosaccharide probes) in a total of 450 mL water (Marchbank et al. 2011). The participants remained rested and fasted during the collection period. However, they were allowed unlimited intake of water to ensure adequate urine output. Urine was collected, stored at 4 °C, and pooled over the 2 h following the ingestion of sugar-probe solution. At the end of the collection period, participants were required to empty their bladders once more, following which the total volume of urine was recorded. Aliquots were centrifuged (405×*g* for 15 min at 4 °C) to remove gross debris, and the supernatant was frozen at − 80 °C until analysis.

### Measurements of urinary saccharides by LC–MS/MS

For urinary analysis, acetonitrile (HPLC gradient grade ≥ 99.9%), ammonium formate (Fluka Analytical for HPLC ≥ 99.0%), lactulose (≥ 95%), mannitol and rhamnose (≥ 99%) were supplied by Sigma-Aldrich (Auckland, New Zealand). Internal standards, ^13^C_6_ mannitol and ^13^C_12_ lactulose, were supplied by Santa Cruz Biotechnology (Dallas, Texas, USA). Type I water was prepared using a Milli-Q Reference A+ ultrapure water purification system (Merck, Auckland, New Zealand) with a resistivity of 18.2 MΩ.

Urinary saccharide concentrations were measured by high-performance liquid chromatography-tandem mass spectrometry based on methods previously described (Kubica et al. 2012; Lee et al. 2014; Lostia et al. 2008; Musa et al. 2019). Samples were thawed at 4 °C, vortexed and centrifuged (Eppendorf 5810R; 4000×*g*, 4 °C, 10 min). An aliquot (40 µL) was transferred to an amber glass HPLC vial equipped with a 200 µL glass vial insert and amended with internal standards (20 µL, 25 µg/mL) and 90% acetonitrile (140 µL). Pooled quality control (QC) samples were prepared similarly following transferring an aliquot of each sample into a pooled composite sample.

A 7-point calibration set was prepared from a mixed stock solution to give calibration ranges of 0.2–100, 2–1000, and 0.1–50 µg/mL for rhamnose, mannitol, and lactulose, respectively, with ^13^C internal standards at 2.5 µg/mL.

Liquid chromatography-tandem mass spectrometry (LC–MS/MS) was carried out using a Thermo Scientific Accela HPLC system coupled to a Q Exactive Orbitrap mass spectrometer with heated electrospray ionisation (HESI) (Thermo Scientific, New Zealand). Samples were introduced to the HPLC system via a PAL autosampler (injection volume = 5 µL), and chromatography was performed using a Phenomenex Luna NH2 column (2 × 150 mm, 3 µm, Phenomenex, New Zealand) with a gradient elution of type I water with 1 mM ammonium formate (eluent A) and 90% ACN with 1 mM ammonium formate (eluent B) at a flow rate of 300 µL/min. The gradient elution program was as follows: 100% B (0–0.5 min), 100–85% B (0.5–3.5 min), 85% B (3.5–11 min), and a re-equilibration period at 100% B for 3 min (11–13 min). The first 1.5 min of the chromatographic run was diverted to waste and data were acquired from 1.5 to 11 min. The mass spectrometer was operated in negative ionisation mode and data were acquired in full scan MS1 mode and parallel reaction monitoring (PRM) mode for all samples. Full scan data were collected with a scan range of 50–500 *m*/*z*, AGC target of 10^6^, and maximum IT of 100 ms. PRM mode was operated with the inclusion list and relevant parameters listed in Table 1. HESI source settings were as follows; spray voltage of 4.5 kV, capillary temperature of 350 °C, and gas flow rates of 20, 10, and 5 arbitrary units for sheath, aux, and sweep gas, respectively.

**Table 1:** LC–MS/MS PRM inclusion list

Name	Isolation mass (*m*/*z*)	Isolation width (*m*/*z*)	Adduct	Start (min)	End (min)	(N)CE
Rhamnose	163	2	[M-H]^−^	1.5	11	30
Mannitol	181	2	[M-H]^−^	1.5	11	40
^13^C_6_ mannitol	187	2	[M-H]^−^	1.5	11	40
Lactulose	341	2	[M-H]^−^	1.5	11	30
^13^C_12_ lactulose	353	2	[M-H]^−^	1.5	11	30

Prior to sample analysis, method validation was carried out across 3 days to assess intra and interday analyte recovery and variation. Urine samples were spiked with mannitol and lactulose at 3 concentration levels (40, 200 and 500 µg/mL) in triplicate for each validation day and marginal recovery was calculated relative to unspiked urine samples from the following equation:

$$\mathrm{\%recovery}= \left(\frac{{C}_{{\text{m}}}-{C}_{{\text{u}}}}{{C}_{{\text{s}}}}\right)\times 100$$

where C_m_ is the measured spiked sample concentration, C_u_ is the measured unspiked sample concentration, and C_s_ is the actual spiked concetration.

Recovery across days was compared by ANOVA, with *P* > 0.05 indicating no significant difference in recovery percentage between days.

Samples were randomised after assigning a random number to each sample using Excel spreadsheet software (Microsoft) and sorted to give the sample run order in which they were loaded into the autosampler. Pooled QC samples were injected every 10 samples, and technical QC standards were injected every 20 samples. Calibration standards were run at the beginning and end of the batch. Calibration fit, QC repeatability and analyte recovery are given in Table 2.

**Table 2:** Analyte calibration and repeatability metrics

Analyte	Calibration fit (*R*^2^)	Pooled QC (% RSD)	Technical QC (% RSD)	Recovery (%)	Recovery (% RSD)
Rhamnose	0.998	6.7	5.9	–	–
Mannitol	0.999	5.8	2.6	96.0	4.0
Lactulose	0.999	5.4	1.3	94.0	6.8

### Intestinal permeability measurements

Percent recovery of each sugar in the urine and their ratio was assessed based on the calculations described by Musa et al. (2019). Briefly, the amount of each sugar excreted (mg) was calculated by multiplying the concentration of sugar in the urine sample (mg/mL) by the total volume of urine excreted over the 2-h collection period (mL). The percent recovery (%) was calculated by dividing the amount of sugar excreted (mg) by the amount of sugar consumed by the participant immediately prior to the collection period (mg) and multiplying by 100. Finally, the lactulose/rhamnose (L/R) and lactulose/mannitol (L/M) recovery ratios were calculated by dividing the lactulose percent recovery (%) by the rhamnose percent recovery (%) or the mannitol percent recovery (%), respectively.

### Statistical methods

Statistical analysis was performed using R (R Core Team 2023) (version 4.3.1), and data were plotted using the ggplot2 R package (Wickham 2016). For physiological responses for exercise (heart rate, RPE, and *V*O_2_), data were fitted using a mixed model with trial + treatment + time + treatment × time as fixed effects, and participant as a random effect. Estimated marginal means and their standard errors were calculated using the emmeans (Lenth 2023) package in R. A similar method was used for exercise intensity, with trial + treatment + treatment × trial as fixed effects, and participant as a random effect. L/R and L/M and recovery ratios were log transformed for analysis. When testing for carryover effects in the baseline ratios, data were fitted using the model first treatment + trial + first treatment × trial as fixed effects, with participant as a random effect, where ‘first treatment’ was the treatment allocated in the first trial arm. Effect of treatment on exercise-induced gut permeability was analysed using the model sex + age + treatment + exercise + treatment × exercise as fixed effects and participant as a random effect.

## Results

### Participant characteristics

**Fig. 2:**
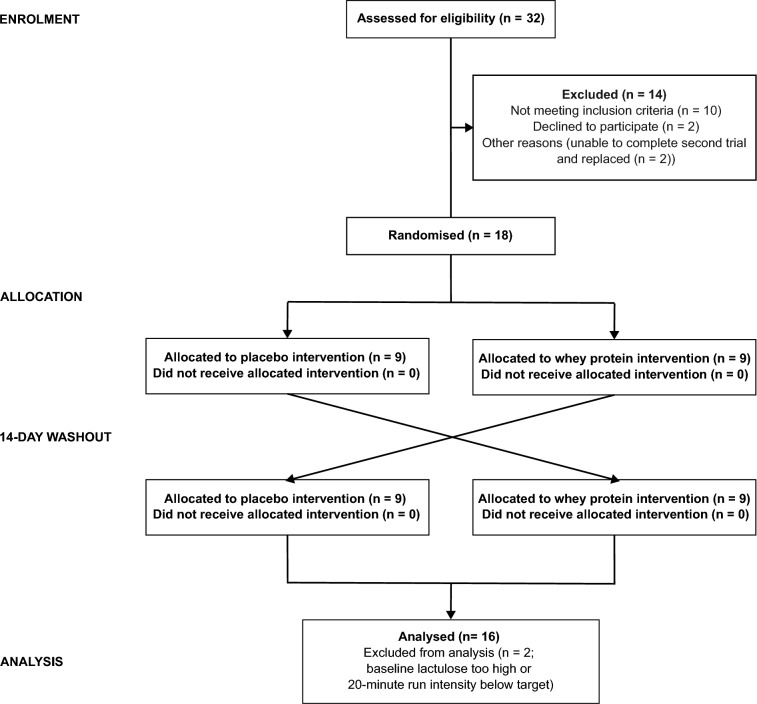
Consolidated standards of reporting trials flowchart for a randomised trial

Thirty-two participants were screened for eligibility, but 10 did not meet the eligibility criteria (Fig. 2). Of the remaining, two participants declined to participate, and a further two were unable to complete both arms of the trial due to injury or illness. Although 18 participants completed both arms of the study, one participant was not able to maintain the required intensity during one of the exercise trials, while an overly high level of baseline lactulose was detected during one of the trial arms for another participant. Hence, 16 participants (eight males and eight females) were considered for analysis. The mean ± SEM age, weight, and height, for males and females, respectively, was 27.5 ± 3.6 years, 71.8 ± 2.8 kg, and 179 ± 2.0 cm, and 32.6 ± 4.3 years, 59.4 ± 2.0 kg, and 168 ± 3 cm. The mean ± SEM $\dot{V}$O_2 max_ was 54.1 ± 1.5 mL/kg/min for males and 45.0 ± 1.2 mL/kg/min for females.

### Physiological and perceptual responses to exercise

**Fig. 3:**
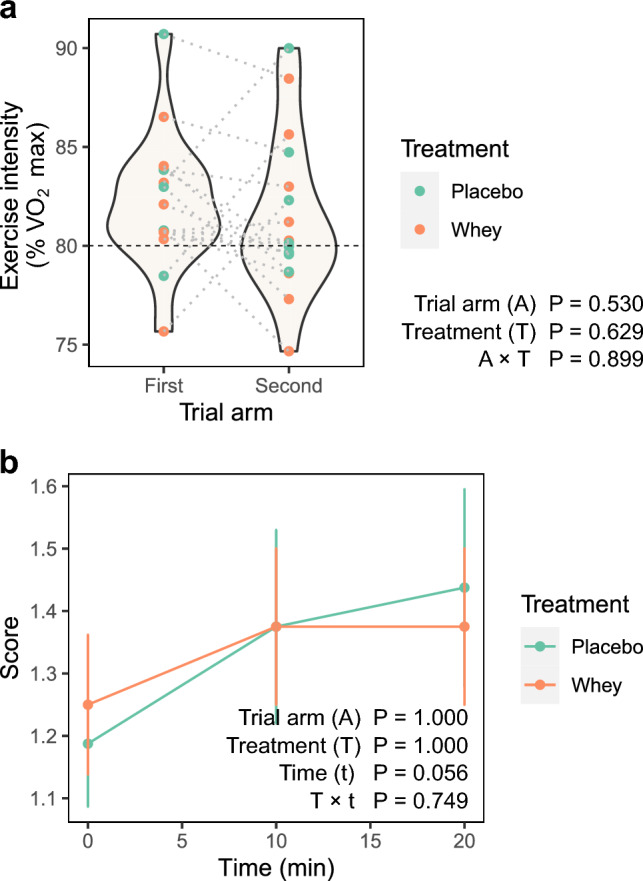
Exercise and responses to exercise. **a** Intensity of exercise during day 14 exercise trial. Each dot represents the running speed of a given participant as the % of their $\dot{V}$O_2 max_ in the given arm. The colour of the dot represents the treatment allocated to the participant in each arm. The violin plot shows the distribution of the data. **b** Gut discomfort over time by treatment. Graph shows mean (± SEM) gut discomfort scores immediately prior to (0 min) and at 10 and 20 min during the run. The gut discomfort scores are 1 (none), 2 (slight), 3 (mild), 4 (moderate), 5 (moderately severe), 6 (severe), and 7 (very severe)

Average running speed was 82.2 ± 1.7% of $\dot{V}$O_2 max_ during the first exercise trial, and 81.5 ± 2.0% of $\dot{V}$O_2 max_ during the second exercise trial (Fig. 3a). The heart rate, RPE and $\dot{V}$O_2_ responses to exercise did not differ significantly between the treatment arms (Table 3). Heart rate, RPE and $\dot{V}$O_2_ increased over time, similarly, during exercise for both treatment arms.

**Table 3:** Physiological responses to exercise

	5 min	10 min	15 min	20 min	Anova
*V*O_2_ (L/min)					
Placebo^a^	2.61	2.70	2.72	2.74	Trial *P* = 0.734 Treatment (*T*) *P* = 0.588 Time (*t*) *P* < 0.001 *T* × *t* *P* = 0.932
Whey^a^	2.60	2.74	2.74	2.75	
SED^b^	0.0513	0.0513	0.0513	0.0513	
* P* value^b^	0.851	0.458	0.765	0.818	
Heart rate (bpm)					
Placebo	154	161	163	165	Trial *P* = 0.452 Treatment (*T*) *P* = 0.055 Time (*t*) *P* < 0.001 *T* × *t* *P* = 0.988
Whey	156	162	165	167	
SED^a^	2.27	2.27	2.27	2.27	
* P* value^a^	0.209	0.443	0.367	0.352	
RPE					
Placebo	10.0	11.0	11.8	12.4	Trial *P* = 0.580 Treatment (*T*) *P* = 0.331 Time (*t*) *P* < 0.001 *T* × *t* *P* = 0.619
Whey	10.1	11.2	11.7	12.0	
SED^a^	0.365	0.365	0.365	0.365	
* P* value^a^	0.798	0.496	0.798	0.269	

^a^Values are estimated marginal means (from the mixed model with trial + treatment × time as fixed effects and participant as a random effect)

^b^Standard error of the difference (SED) of estimated marginal means at each timepoint and respective *P* value

All participants reported having no or only slight gut discomfort (score of 1–2 in the gut discomfort scale) before and during the run, with the exception of one participant allocated the placebo reporting mild gut discomfort (score of 3) during the second arm. Gut discomfort scores trended upwards over time (*P* = 0.056), however there was no treatment or treatment × time effect (Fig. 3b). Following the run, one of the participants in the whey arm reported their ‘stomach feeling bloated’, while another reported ‘passing gas’. In the placebo arm, one participant reported ‘hunger pains in the stomach’. Two other participants (one from each trial arm) reported ‘rumbling’. None of the participants reported symptoms of ‘stomach-ache or pain’, ‘heartburn’, ‘acid reflux’, ‘hunger pains in stomach’, ‘nausea’, ‘burping’, ‘urgent bowel movement’, or ‘urgent need to urinate’.

### Intestinal permeability

**Fig. 4:**
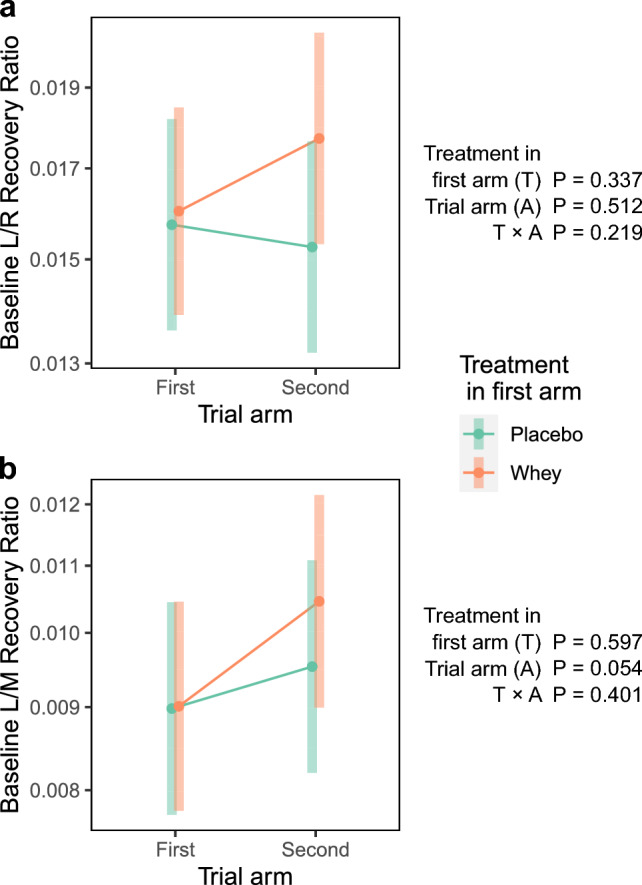
Testing for carryover effects. Graphs show the estimated marginal means and 95% confidence intervals for **a** baseline lactulose/rhamnose, and **b** baseline lactulose/mannitol recovery ratios

Baseline L/R values were similar at the beginning of the first and second trial arm. However, baseline L/M values trended to be 11 ± 6% higher at the start of the second arm compared to the first arm (*P* < 0.1). To determine whether there was a carryover effect of treatment between the two arms of the study, we compared the baseline L/R and L/M values of participants who were assigned placebo in the first arm against those assigned whey in the first arm (Fig. 4). The second trial had higher baseline L/M (*P* = 0.054). Participants assigned whey in the first arm had a higher baseline L/M in the second arm compared to the first arm (*P* < 0.05). However, there was no interaction between treatment in the first arm and the trial arm (*P* = 0.4) indicating there was no carryover effect. Similarly, no carryover effect could be observed when comparing the L/R baseline values (*P* = 0.3; Fig. 4).

**Fig. 5:**
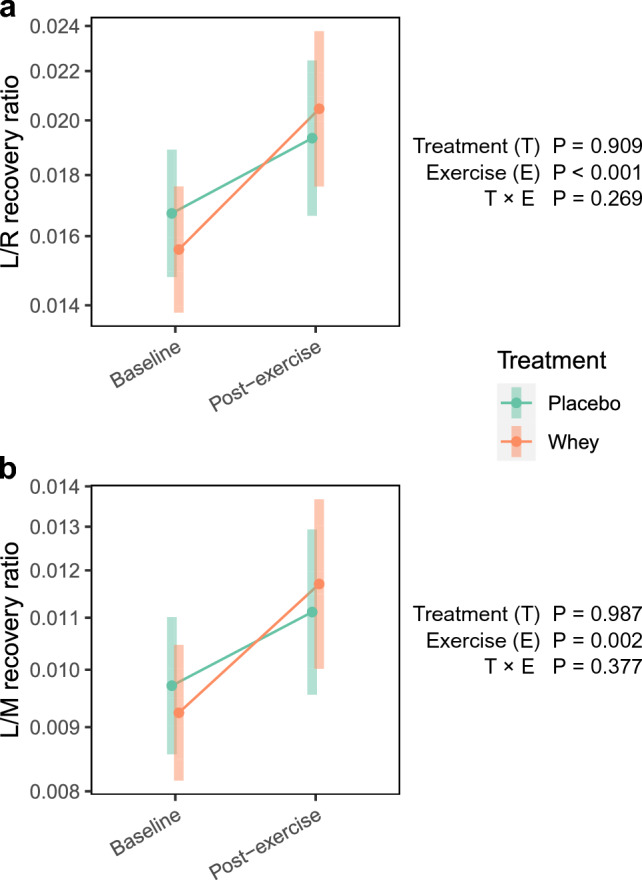
Effect of treatment on exercise-induced gut permeability. Graphs show the estimated marginal means and 95% confidence intervals for **a** lactulose/rhamnose, and **b** lactulose/mannitol recovery ratios

Gut permeability increased in response to exercise; the L/R recovery ratio increased by 23 ± 7% (*P* < 0.001), while the L/M recovery ratio increased by 20 ± 7% (*P* < 0.005) (Fig. 5). The sex effect was not significant for either the L/R (*P* = 0.6) or L/M (*P* = 0.7) recovery ratios. There was not strong evidence for an age effect for L/R (*P* = 0.06) or L/M (*P* = 0.1) recovery ratios.

While the increase in permeability in the whey arm (31 ± 14% L/R recovery ratio, and 24 ± 14% L/M recovery ratio) appeared to be higher than in the placebo arm (15 ± 12% L/R and L/M recovery ratios), there was no statistically significant difference in baseline permeability or post-exercise permeability between the arms. Neither the treatment effect, nor treatment × exercise effect, was significant (Fig. 5).

## Discussion

We investigated whether a commercially prepared bovine whey protein ingredient that contains all bovine milk whey proteins with an isoelectric point > 6.8 could mitigate exercise-induced increases in gut permeability. While the majority of whey proteins in bovine milk comprise of β-lactoglobulin (~ 50%) and α-lactalbumin (~ 20%), bovine milk also contains several minor whey proteins with potent bioactivity, albeit at a lower concentration (Smithers 2008). When producing the whey ingredient used in this study, the separation of whey proteins based on an isoelectric point of 6.8, eliminates the major whey proteins thereby increasing the concentration of several minor bioactive whey proteins (Abd El-Salam and El-Shibiny 2017).

In our participant cohort, running at 80% $\dot{V}$O_2 max_ for 20 min at a 1% incline increased gut permeability by 20–23% as measured by L/M and L/R recovery ratios. This increase in gut permeability did not coincide with any gastrointestinal symptoms, and participants reported no or only slight gut discomfort during exercise. This is consistent with previous studies, where acute exercise has been shown to cause gut permeability in the absence of gut discomfort (Keirns et al. 2020). The physiological and perceptual stress was comparable between the trial arms, thus changes to post-exercise permeability between trials can be attributed to the treatment. However, there was no significant treatment × exercise effect, indicating that the whey protein ingredient was no better or worse than the placebo at alleviating the observed increase in gut permeability following exercise.

We opted to assess intestinal permeability by urinary excretion of ingested probes, as this method is widely accepted in the literature, and is less invasive than assessing biomarkers in blood (Schoultz and Keita 2020). A smaller monosaccharide probe represents the constant flux across the intestinal barrier, while an elevated flux of a larger disaccharide probe will lead to an increase in the disaccharide/monosaccharide ratio and is an indicator of loss of barrier function. We used lactulose as the disaccharide probe, and rhamnose and mannitol as two alternative monosaccharide probes in a hypo-osmolar formula, as this formulation had been previously used by others to successfully detect exercise-induced barrier dysfunction (Marchbank et al. 2011; Davison et al. 2016; March et al. 2017). However, analysis of ingested probes in blood has the advantage of being able to detect smaller and transient changes that may not be detected via urinary analysis. This is evident in studies that examined both urinary L/R and plasma L/R, and found that plasma L/R, but not urinary L/R, increases following exercise (van Wijck et al. 2011; JanssenDuijghuijsen et al. 2016). Thus, it is possible that the whey treatment had a subtle or transient impact on exercise-induced gut permeability, which might have been detected should we have analysed blood sampled over time following exercise.

Our observed 23% increase in L/R recovery was very modest compared to other studies which utilised the same exercise protocol, where L/R recovery increased by 250–300% in the placebo arm (Marchbank et al. 2011; Davison et al. 2016; March et al. 2017). However, different studies have shown smaller or statistically non-significant increases in L/R recovery following a bout of exercise. In fact, one study showed a statistically significant reduction in L/R recovery following 90 min of cycling (van Nieuwenhoven et al. 2004). In another study, cycling after having consumed ibuprofen was shown to increase L/R recovery compared to when at rest after consuming ibuprofen (Van Wijck et al. 2012b). However, this increase could not be observed when the participants did not consume ibuprofen. This discrepancy between studies is likely due to various factors that can contribute to the degree of L/R recovery following exercise. For example, the level of permeability is dependent on the intensity at which exercise is carried out, with exercise at 80% $\dot{V}$O_2 max_ shown to induce gut permeability significantly more than exercise at lower intensities (Pals et al. 1997). In addition, the duration of exercise, the environment, the participant’s core temperature, and the timing of ingestion of the sugar probes have all been shown to contribute to post-exercise urinary probe recovery (Keirns et al. 2020; Chantler et al. 2021). While an acute bout of exercise can increase gut permeability, exercise in the long-term is shown to benefit gut barrier integrity (Keirns et al. 2020). Thus, it is plausible that normal exercise regime of the trial participants may also impact on their post-exercise gut permeability levels.

Our study employed the same exercise protocol, probe solution, and timing of probe ingestion as the studies which observed a 250–300% increase in L/R (Marchbank et al. 2011; Davison et al. 2016; March et al. 2017). However, while the previous studies performed a 5-h urine collection, we opted for urine collection over 2 h. The timing of urine collection is dependent on the region of interest within the gastrointestinal tract. Using concurrently administered radioisotopes and oral sugars, it has been established that urine collections from 0 to 2 h are reflective of the small intestinal permeability, while urine samples collected from 5 to 24 h reflect colonic permeability (Rao et al. 2011). As whey protein is highly digestible, and thus absorbed in the upper gastrointestinal tract, the dietary intervention used in our study is more likely to exert its effects on the small intestine rather than the colon (Sousa et al. 2023). Moreover, a study comparing 2-h vs 5-h urine collections showed that although recovery of lactulose and mannitol was higher in the 5-h collection, there was no difference in the L/M ratios between the two collections (Musa et al. 2019). Hence, it is unlikely that the difference in exercise-induced increase in permeability is solely due to difference in timing of urine collection. It should be noted, however, that while the timing of urine collection in our study was based on Musa et al. (2019), a study by Sequeira et al. (2014) recommended that clinical tests of small intestinal permeability are restricted to 2.5–4 h post dosage, as during this time window the inter-subject variation in sugar excretion is minimised. Thus, delaying the urine collection by 0.5–2 h could be considered for future studies.

Another key difference in our study was the inclusion of female participants. Previous studies which observed a 250–300% increase in L/R (Marchbank et al. 2011; Davison et al. 2016; March et al. 2017), and in fact most studies which assessed exercise-induced gut permeability via urinary probes (Chantler et al. 2021), utilised male-only participant cohorts. We speculate that this may be due to the added complexity of sampling urine from females and accounting for the effects of the menstrual cycle on gut permeability (Roomruangwong et al. 2019). We recruited equal numbers of male and female participants to ensure our study was representative of the population. Previous findings have indicated that biological sex has no effect on exercise-induced intestinal permeability when females are in the follicular phase of the menstrual cycle (Snipe and Costa 2018), as was the case in our study. Consistent with previous studies, we did not detect a difference in exercise-induced gut permeability between male and female participants. Hence, the inclusion of both male and female participants is unlikely to have confounded our findings.

While we did not detect an effect of the dietary treatment in our study, dietary compounds are recognised as playing an important role in gut barrier modulation. Certain food components such as gluten, ethanol, and emulsifiers in processed foods are known to increase intestinal permeability (Khoshbin and Camilleri 2020). Conversely, vitamins and amino acids, as well as short-chain fatty acids derived from dietary fibre, reduce intestinal permeability. Bovine milk and dairy products, such as that used in the present study, are rich in nutrients and bioactive components that can promote gut health (Park and Nam 2015). In particular, lactoferrin (Playford et al. 2001) and colostrum (Anderson et al. 2019) have been shown to improve intestinal barrier integrity. In fact, colostrum supplementation is being recognised as a strategy to improve intestinal permeability in athletes (Dziewiecka et al. 2022). Lactoferrin is a key constituent of colostrum, but colostrum also contains high levels of other immunological compounds (including immunoglobulins, lactoperoxidase), growth factors, vitamins, and nutrients (including omega-3 and -6 fatty acids, and short-chain fatty acids). Although the whey protein ingredient used in our study was rich in bioactive proteins such as lactoferrin and lactoperoxidase, the potential of colostrum to mitigate exercise-induced gut permeability as reported elsewhere (March et al. 2017; Marchbank et al. 2011) may have been due to a combined effect of these bioactive proteins with the other constituents of colostrum. However, dosage may also play a role. In our study, participants were required to consume 200 mg/day of whey protein, as this dose is in line with the recommended daily intake, and has previously been shown to exert immune responses in the gut (Quantec Ltd., personal communication). In the studies where the effects of colostrum were investigated, the treatment was consumed at 20 g/day (March et al. 2017; Marchbank et al. 2011), 100-fold higher than the dose of whey protein consumed in our study. Thus, a higher dose of the whey protein ingredient may have had an effect on gut permeability following exercise.

The whey protein ingredient has previously been shown to improve barrier integrity in ‘healthy’ intestinal epithelial monolayers in vitro, and mitigate tumour necrosis factor alpha (TNF-α)-induced loss of barrier function in vitro (Ulluwishewa et al. 2022). The in vitro findings do not contradict the observations of the present study because (i) in this study we did not investigate the effect of whey protein on ‘healthy’ (pre-exercise) gut permeability, and (ii) exercise-stress does not cause barrier defects via the same mechanisms as TNF-α (Hering et al. 2012). Moreover, while the in vitro measures were carried out in the presence of the whey protein ingredient, in the present study, participants carried out the exercise trial following an overnight fast. Hence, whether the timing of delivery of the whey ingredient to the intestine can impact the exercise-induced barrier dysfunction is yet to be determined. It should be noted that the in vitro studies did not consider factors such as temperature, oxidative stress, oxygen levels, or digestion of the whey protein ingredient, among others, that could influence the in vivo exercise response (Lambert 2008; van Wijck et al. 2012a; Sultan et al. 2018). Thus, further in vitro and in vivo research is needed to understand the potential of the whey protein ingredient as a functional food to improve intestinal barrier integrity. Future research should investigate the effect of dose on various measures of gut barrier function (e.g. blood biomarkers, and plasma L/R ratios in addition to urinary L/R ratios) under unstressed (e.g. pre-exercise) as well as stressed (e.g. post-exercise) states. Investigation of rate of recovery of barrier function following stress, and the effect of the timing of delivery of whey, may also provide further insights. In vitro investigations could provide mechanistic insights, such as the effect of the whey ingredient on temperature, and oxidative stress-induced responses in intestinal cells, without confounding factors such as blood flow and gut hormones. A better understanding of the effects of natural ingredients, such as those derived from dairy, will enable the development of targeted strategies to improve gut barrier function using food.

## Data Availability

The data supporting the conclusions of this article can be made available by the authors, upon reasonable request.

## Abbreviations

bpm: Beats per minute
L/M: Lactulose/mannitol
L/R: Lactulose/rhamnose
PRM: Parallel reaction monitoring
RPE: Rating of perceived exertion
SEM: Standard error of the mean
$\dot{V}$O_2 max_: Maximal oxygen uptake
